# Novel MSC Perspectives: From Cell Regulation to Tissue Regeneration

**DOI:** 10.3390/ijms241713392

**Published:** 2023-08-29

**Authors:** Heba Abdelrazik, Alessandra Pelagalli

**Affiliations:** 1 Department of Immunology and Translation Medicine, University of Genoa and San Martino Hospital, 16128 Genova, Italy; 2 Department of Clinical Pathology, Immunology and Cell Therapy Unit, Cairo University, 12613 Cairo, Egypt; 3 Department of Advanced Biomedical Sciences, University of Naples Federico II, Via Pansini 5, 80131 Naples, Italy; alpelaga@unina.it; 4 Institute of Biostructures and Bioimages, National Research Council, Via De Amicis 95, 80131 Naples, Italy

Three Special Issues, so far, have been dedicated to overall MSC prospective biology, from cell regulation to tissue regeneration. This third Special Issue contains 9 research articles and 11 reviews which tackle innovative discoveries aiming to improve the utilization of MSC and their extracellular vesicles (EVs) not only for therapeutic approaches and clinical protocols but attempts to overcome some of the current limitations encountered by MSC (Figure 1). In this regard, an anti-let-7 miRNAs antisense oligonucleotide combination was discovered to increase MSC proliferation in vitro. The combination delayed MSC senescence while increasing the self-renewal of neighboring hematopoietic progenitors and, more importantly, without the additional acquisition of stemness, thus moving a step further towards safer and more efficient MSC production.

The use of fetal bovine serum (FBS) in MSC expansion and propagation protocols is indeed another limitation with major concerns. Two research articles addressed the use of xeno-free conditions. One study evaluated ultracentrifugation and ultrafiltration to deplete MSC-EV from the contaminating proteins of FBS. Indeed, serum had a positive effect on EV, the depletion of which affected MSC immunomodulatory capacity, so a switch to human serum was advised. Meanwhile, the other study used xeno-free three-dimensional microcarrier culture systems, which also potentiated MSC anti-inflammatory capacity due to a better suppression of TNF-α and IL-1. Together, they have garnered increasing interest to the potential mass production of safe, zoonotic transmission-free MSC.

Major MSC regulatory factors, TNF-α and IFN-γ, together with the effects of cell-to-cell contact and EVs, were reviewed. Due to the heterogeneity of the effects, the design of in vitro activation protocols to improve the immunoregulatory activity of MSC has been proposed. Accordingly, another article demonstrated a protocol to transduce MSC with superoxide dismutase 3 protein as an antioxidant in order to protect the intestinal cells or organoids from inflammation-mediated epithelial damage through the downregulation of inflammatory responses, as well as the maintenance of epithelial junctional integrity.

Not missing from the pandemic scene, MSC may play a crucial role as they can modulate the cytokine storm [1] and the long-term immunological concerns caused by COVID-19 and long-COVID-19 due to their two-way immunoregulation and regenerative capabilities. A review article demonstrated the clinical trials conducted so far; the authors concluded that MSC can be a standalone low-cost alternative compared to the current protocols via a combination of different types of drugs that are not cost effective for patients.

An interesting review highlighted the mechanical-induced forces on the fate of MSC and how the cells perceive and react to their mechanical environment. Due to fluid shear stress, cells are exposed to a variety of mechanotransduction, a complex mechanism interconnecting stem cells with their physical environment, influencing their fate and biological activity. This may explain some of the heterogeneity within the results of MSC, as well as their extensive properties. Indeed, cells were able to form vascular tube-like structures when co-cultured with living cardiac tissue, yet they failed to do the same with ischemic tissues due to the absence of shear forces [2]. Moreover, another contribution confirmed the aforementioned results, stating that the transition from adhesive to spheroid MSC increased the expression of VEGF and its receptor on MSC, and thus their angiogenic potential.

A systematic review utilizing 24 studies evaluated the effect of hypoxia during expansion, and the chondrogenic differentiation of MSC was also included. The review demonstrated inconsistent results and conflicting reports that were explained by the heterogeneity of the studies involved. The authors recommended the standardization of the techniques used, yet the aforementioned effect of the microenvironment and physical stress may also play a role. Cambré et al. revealed that MSC converts mechanical stimuli into chemical stimuli, causing local inflammation and bone destruction. Shortly thereafter, MSC was stimulated by the microenvironment, inducing bone formation and differentiation. Moreover, MSC can change the response from M1 to M2 following compression [3].

A couple of studies investigating novel MSC differentiation protocols were included in this Special Issue. Rather than genetic transduction, targeted epigenetic modification was approached. A class II histone deacetylase (HDAC) inhibitor, “MC1568”, amplified and accelerated the formation of osteocytes in about 7 days for the replacement of bone tissue, a much shorter time frame than that that normally observed, which is around 21 days. In addition, β-mercaptoethanol or basic fibroblast growth factor (bFGF) pre-induced MSC were used to coat cellulose/collagen nanofibrous nerve conduit for facial nerve regeneration in a rat model. The latter showed the highest degree of recovery based on functional and histological evaluations compared to non-pre-induced MSC and non-coated fibers. Furthermore a study demonstrated the biocompatibility of the novel biomorphic B-HA scaffold and its potential use in the osteogenic differentiation of MSC, yielding tissue regeneration without the aid of additional growth factors, bioactive molecules, nor cells. These results could have a significant impact on the translational processes, avoiding regulatory complexes linked to the use of osteogenic molecules, thus reducing time and cost.

Addressing dentistry medicine, this issue included a review on the dental stem cell family which could benefit major dental branches like endodontics, oral surgery, and periodontics, providing less invasive (more conservative) treatment to patients.

To overcome MSC heterogeneity, EVs, also hereby called exosomes or secrotomes, have emerged as a novel source responsible for a vast majority of MSC therapeutic potentials. EVs are able to repair damaged tissues, regulate the immune response, and reduce inflammation. EVs have advantages over MSC, including small dimensions, low immunogenicity, and no need for additional procedures for expansion. Some of the contributions herein outlined their pivotal roles, which reformulated our knowledge of MSC potentials; EVs as a cell-free platform for therapeutic drug delivery could control tumor progression, including, but not limited to, brain tumors as they are able to cross biological barriers. Moreover, engineering EVs to become next-generation nanocarriers; packaging drugs and genes into EVs acting as therapeutic drug delivery vehicles for cancer treatment has been reviewed. Others have reviewed EVs potentials for functional recovery after spinal cord injury, utilizing their ability to reduce inflammation for age-related musculoskeletal frailty (sarcopenia) through their capacity to inhibit muscle cell apoptosis and activate muscle stem cells, in addition to their possible use in osteoporosis as an alternative or adjunctive to biphosphonates. Interestingly, it was stated that EVs can regulate metabolic processes, so they could be potential next-generation anti-obesity agents and even be used in metabolic syndromes.

For the translation of the abovementioned therapeutic potentials, one study investigated the capacity of EVs, formulated as a ready-to-use and freeze-dried medicinal product (the Lyosecretome), to promote the osteoinductive and osteoconductive properties of titanium cages, which can be easily adapted by the clinical community for bone regeneration. These clinical applications undoubtedly open up a fascinating scenario for the development of a new generation of EVs applications. However, although the safe/appropriate use of EVs in clinical practice appears feasible and tangible, standardized good manufacturing practices (GMPs) for the clinical grade production of EVs is needed for the optimization of cell cultures, purification, quantification, and quality control.

All together, these articles paint a picture of an orchestrated web of signals and interactions that can be modulated to impact advances in MSC translation to reach the goal of using MSC in routine clinical practice. Currently, the available data point to the need for stem cell precision medicine; moreover, a case-by-case evaluation of MSC clinical applications is required, particularly in relation to donor characteristics, specific culture conditions, and specific therapeutic applications. Stem cell precision medicine focuses on creating and applying 3D, patient-specific disease models and is worthy of further research [4].

## Figures and Tables

**Figure 1:**
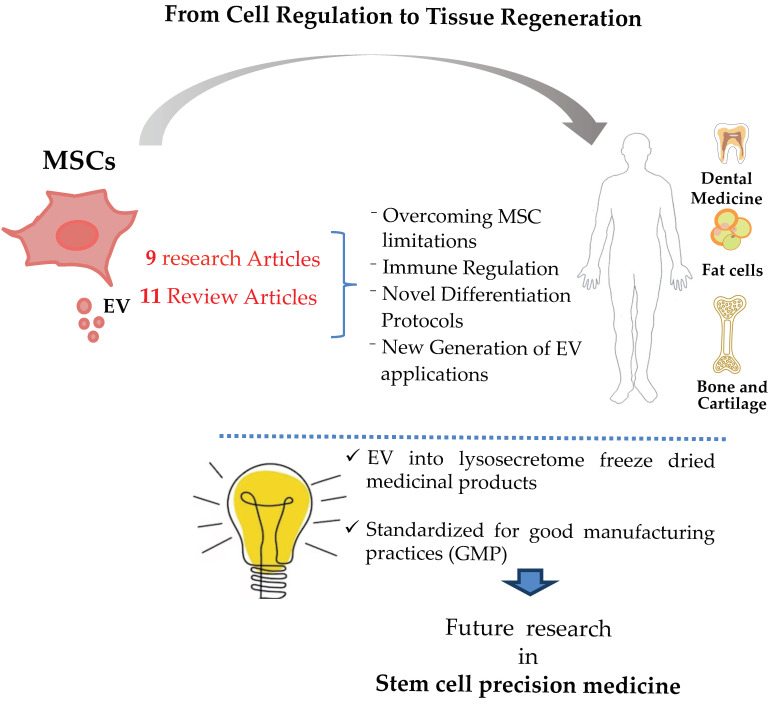
Overview of the topics addressed in our third Special Issue.
